# All-Electron Plane-Wave Electronic Structure Calculations

**DOI:** 10.1021/acs.jctc.2c01191

**Published:** 2023-02-09

**Authors:** François Gygi

**Affiliations:** Department of Computer Science, University of California Davis, Davis, California 95616, United States

## Abstract

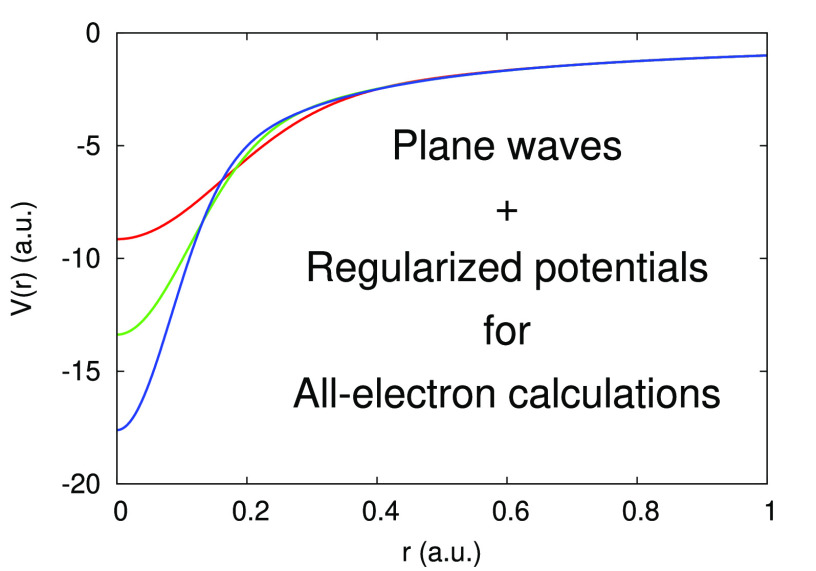

We demonstrate the use of the plane wave basis for all-electron electronic structure calculations. The approach relies on the definition of an analytic, norm-conserving, regularized Coulomb potential, and a scalable implementation of the plane wave method capable of handling large energy cutoffs (up to 80 kRy in the examples shown). The method is applied to the computation of electronic properties of isolated atoms as well as the diamond and silicon crystals, MgO, solid argon, and a configuration of 64 water molecules extracted from a first-principles molecular dynamics simulation. The computed energies, band gaps, ionic forces, and stress tensors provide reference results for the validation of pseudopotentials and/or localized basis sets. A calculation of the all-electron band structure of diamond and silicon using the SCAN meta-GGA density functional allows for a validation of calculations based on pseudopotentials derived using the PBE exchange-correlation functional. In the case of (H_2_O)_64_, the computed ionic forces provide a reference from which the errors incurred in pseudopotential calculations and in localized Gaussian basis sets calculations can be estimated.

## 1 Introduction

The importance of density functional theory^1^ as an electronic structure method has motivated the development of many numerical electronic structure computation methods during the past decades. With the growing need to validate approximations used in DFT electronic structure theory, in particular the choice of exchange-correlation functional, more attention has been paid to the accuracy of the numerical methods used in the solution of the Kohn–Sham (KS) equations^2^ of DFT. The validation of a physical approximation (such as, e.g., the choice of a specific exchange-correlation functional) requires the ability to increase numerical accuracy to the point where numerical errors are much smaller than the differences due to physical approximations.

A comprehensive review of electronic structure methods has been given by Martin.^3^ Among the many existing electronic structure methods, all-electron methods, i.e., including both core and valence electrons, must address the inherent multiscale character of the electronic structure problem that stems from the range of length and energy scales involved in the description of core and valence electrons. For an element of atomic number *Z*, the ratio of length scales involved is directly proportional to *Z*, while the energies involved span a range scaling as *Z*^2^. This multiscale aspect has motivated multiple approaches that aim to include both core and valence electrons. Such methods include augmented plane wave (APW) methods,^4^ multiresolution methods,^5,6^ numerical atomic orbital (NAO) basis methods,^7^ Gaussian basis set methods,^8^ and finite element (FE) methods.^9^ Most of these approaches rely on the use of atom-centered basis functions, either directly in NAO and Gaussian basis methods, through the definition of “muffin-tin” (MT) regions in APW methods, through local adaptive refinement in multiresolution and FE methods, or through enrichment of the basis set by an appropriate choice of localized basis functions in FE methods.^10^

For atom-centered basis sets such as Gaussian basis sets, the process of approaching a complete basis involves the addition of diffuse basis functions as well as higher order spherical harmonics. For example, Lee et al.^11^ have recently shown that large, uncontracted Gaussian-type orbital (GTO) basis sets can achieve a high accuracy in periodic solids. MT-based methods require the addition of localized orbitals in the basis of augmented plane waves (LAPW-lo).^12^ Grid-based methods require a systematic increase of the basis set size, achieved, e.g., by reducing the grid spacing or using higher order finite elements.

Among the above methods, the FLAPW method is currently considered the “gold standard” of all-electron electronic structure methods. Recently, Gulans et al.^13^ have demonstrated convergence of the DFT LDA energy of the order of 1 microHartree (μHa) in the G2–1 set of molecules^14^ using the EXCITING LAPW+lo program.^15^ Multiresolution methods have also demonstrated a similar level of accuracy.^13^ However, approaching the complete basis limit using the FLAPW method still requires expert knowledge, e.g., of the atomic electronic structure in order to define the local orbital basis at appropriate energies, choosing parameters such as the MT radius and angular momentum cutoffs, and controlling the linearization error.^16^ Furthermore, the implementation of the method for recent (meta-GGA) exchange-correlation functionals remains challenging.^17^

In a different context, when used with pseudopotentials, the plane wave method presents a number of attractive features, such as the ability to compute ionic forces without basis set superposition effects, and an unbiased description of unoccupied orbitals. It avoids the issue of overcompleteness of the basis set that affects all localized orbital methods when reaching the complete basis set limit. The translational invariance of the plane wave basis simplifies the computation of the stress tensor of a solid, which in turn allows for straightforward iterative optimization of unit cell parameters.^18^ Numerical implementations also benefit from the availability of efficient Fast Fourier Transform algorithms and efficient reciprocal space preconditioners.

The use of a plane wave basis, however, requires using smooth potentials. Traditionally, this was achieved by using pseudopotentials that remove core electrons and are thus unable to address the all-electron problem directly. This in turn shifts the focus of a high-accuracy calculation on the availability of accurate pseudopotentials. A wide variety of pseudopotentials have been proposed over the past decades, including norm-conserving,^19^ ultrasoft,^20^ and projector augmented wave (PAW)^21^ potentials, each addressing the challenge of describing valence electrons accurately in various environments. The use of a pseudopotential always involves an assumption of transferability from an isolated atom to an arbitrary environment. While considerable progress has been made in recent years to develop accurate pseudopotentials,^22^ their accuracy can only be assessed by comparison with all-electron results when available. This comparison is made difficult by the fact that pseudopotentials must be derived for a specific choice of exchange-correlation functional, and should only be used with that same functional. Furthermore, pseudopotential parameters are typically optimized to reproduce accurately ground state properties computed with all-electron methods (e.g., energy vs volume curves for solids). The description of unoccupied electronic orbitals is typically not included in the figure of merit defining a pseudopotential. This in turn requires the all-electron method of reference to be able to provide accurate empty orbitals and eigenvalues, which can be challenging. A careful validation of pseudopotentials is critical for the computation of band gaps, in particular when the computed DFT band gap is the starting point of a more elaborate calculation using many-body perturbation theory.

In this paper, we describe an all-electron, plane wave electronic structure method that reconciles the accuracy of an all-electron method with the simplicity of the plane wave basis. The method relies on the definition of an analytic, norm-conserving (ANC) potential representing electron–ion interactions, and a scalable implementation of the plane wave method capable of operating with very large energy cutoffs (up to 80 kRy in the examples presented). We show how the electronic structure of atoms and solids can be obtained, and how results can serve as reference data for the validation of pseudopotentials or for the validation of all-electron methods. The method also allows for the computation of the stress tensor, which can be difficult to obtain with typical all-electron methods. Conventional preconditioning approaches used in plane wave implementations can be used without modification at large energy cutoffs, so that the iterative solution of the Kohn–Sham equations does not require the use of specialized algorithms such as, e.g., Chebyshev filtering. We demonstrate the use of the plane wave method in all-electron calculations performed with the SCAN meta-GGA exchange-correlation functional, whose self-consistent implementation in conventional all-electron methods is challenging.^17^ Last, we show how the AEPW calculation of ionic forces in a 64-molecule water system provides reference data that are used to validate pseudopotentials and Gaussian basis sets. We note that other authors have considered the use of regularized Coulomb potentials, such as, e.g., for the smooth representation of electron–electron interactions,^23^ or in the context of multiresolution electronic structure calculations.^5^ The definitions of the regularized potentials used by these authors rely on minimizing errors in the expectation value of the potential energy and differ from the approach we use here.

## 2 Methods and Algorithms

Using the plane wave basis for all-electron calculations depends critically on a careful choice of a smooth potential to replace the electron–ion Coulomb interaction. We define a regularized Coulomb potential that has the following essential properties: (i) it is analytic, i.e., it has no discontinuous derivatives of any order, (ii) it is norm-conserving in the sense defined by Hamann, Schlüter, and Chiang,^19^ and (iii) it depends on a single parameter that can be used to approach the Coulomb potential arbitrarily closely. Analyticity of the potential is necessary to ensure a rapid convergence of a Fourier representation. Discontinuities in the derivatives of a potential cause a slow (algebraic) decay of its Fourier coefficients, which in turn requires the use of a large plane wave energy cutoff in order to reach convergence. The norm conservation condition is critical and ensures that the noninteracting hydrogenoid eigenvalues are accurate and that orbitals are correctly reproduced away from the nucleus. In the case of pseudopotentials, this property was shown to lead to an improved transferability of potentials to arbitrary environments.^19^ Last, a simple parametrization allows for a systematic exploration of the convergence of the method in the limit where the Coulomb potential is recovered.

### 2.1 Analytic Norm-Conserving Regularized Potential

In this section, we first define a regularized potential for the hydrogen atom, and then derive the potentials of all other elements using a simple scaling relation. Starting with the hydrogen atom, we seek to define a smooth analytic potential *V*(*r*) such that

1. The lowest energy solution ϕ(*r*) of the Schrödinger equation
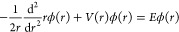
1has eigenvalue
2. ϕ(*r*) is differentiable at *r* = 0
3.

Rather than defining directly a regularized potential, we choose to first define the orbital ϕ(*r*) and then define the potential by inversion of the Schrödinger equation. We use the following definition of the 1s orbital ϕ(*r*)
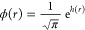
2where the function *h*(*r*) must be defined. Inverting the Schrödinger equation using the known eigenvalue *E*_1*s*_ = −1/2, we obtain
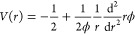
3Using the definition of the function ϕ(*r*) in eq 2, it is easily shown that it satisfies the Schrödinger equation for the potential
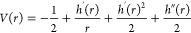
4In order to obtain the correct asymptotic behavior of the wave function ϕ(*r*) as *r* → *∞*, we require
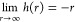
5In order for ϕ(*r*) to be differentiable at *r* = 0, we impose
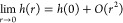
6These conditions are satisfied by the function
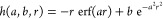
7where *a* is a parameter determining the range of the regularization and *b* is an adjustable parameter. The derivatives appearing in the definition of the potential *V*(*a*, *b*, *r*) are
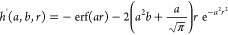
8and
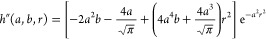
9The potential is finite and differentiable at *r* = 0
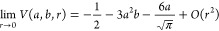
10

#### 2.1.1 Norm Conservation

**Figure 1:**
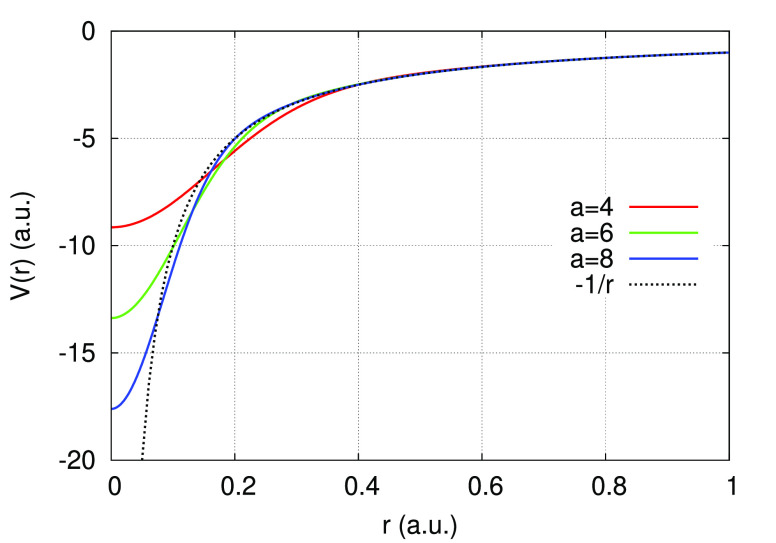
Norm-conserving regularized potential of the hydrogen atom for the values *a* = 4 (red), *a* = 6 (green), and *a* = 8 (blue). The Coulomb potential is shown as a dotted line.

**Figure 2:**
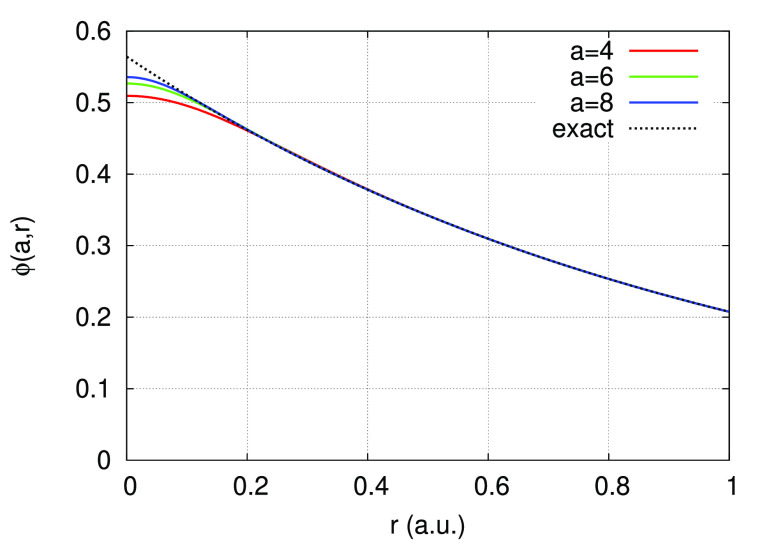
Hydrogen 1s wave function for the values *a* = 4, 6, 8 compared to the exact wave function .

While the potential defined above reproduces the 1*s* eigenvalue exactly by construction, it is important to ensure that higher eigenvalues and corresponding wave functions are reproduced with appropriate accuracy. For that purpose, we impose a norm conservation constraint on the potential, as defined by Hamann, Schlüter, and Chiang.^19^ These authors showed that a norm-conserving potential has enhanced transferability properties, i.e., it is capable of reproducing wave functions accurately at energies that differ from the eigenvalue used in the derivation of the potential. For the specific form of the wave function chosen in eq 2, the norm conservation condition is enforced by adjusting the value of the parameter *b* so that
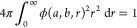
11We note that simply normalizing the wave function by rescaling ϕ(*a*, *b*, *r*) would not satisfy the norm conservation condition since it would modify the wave function at large *r*. Instead, normalizing by adjusting the parameter *b* does not affect the wave function at large *r*. The above expressions thus define a family of analytic, norm-conserving (ANC), regularized potentials that are entirely determined by the choice of the parameter *a*, and approach the Coulomb potential in the limit *a* → *∞*. The parameter *b* is tied to the choice of *a* and is defined by the norm conservation condition. A table of the values of *b* satisfying the norm conservation condition is given in the Supporting Information. In practice, it is found that for the hydrogen atom, values of *a* ≥ 4 yield solutions of the noninteracting Schrödinger equation with 2s, 3s, etc. eigenvalues within a few microHartrees (μHa) of the exact eigenvalues. Increasing the value of *a* reduces the error further below 1 μHa. The regularized potential deviates from the Coulomb potential in a region of radius *O*(1/*a*) near *r* = 0, and the Coulomb potential is recovered in the limit *r* → *∞*. The regularized potential of the hydrogen atom generated using the values *a* = 4, 6, 8 is shown in Figure 1, and the corresponding 1s wave functions are shown in Figure 2.

#### 2.1.2 Convergence of Noninteracting Eigenvalues

**Figure 3:**
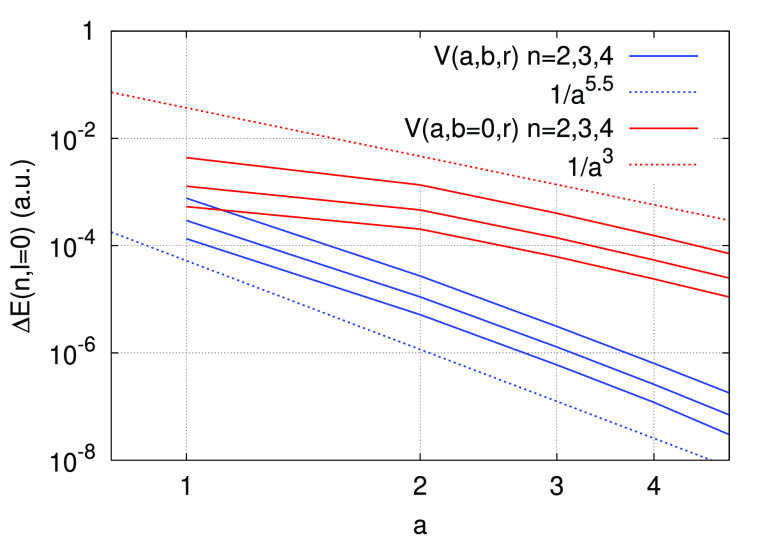
Decay of the error Δ*E*(*n*, *l* = 0) as a function of the parameter *a*, computed with an ANC potential (blue lines) and with a potential *V*(*a*, *b* = 0, *r*) that does not satisfy the norm conservation condition. The top blue line corresponds to *n* = 2 and the bottom blue line to *n* = 4. The order of the red lines is similar. The blue and red dotted lines show the decay of a function ∝1/*a*^5.5^ and ∝1/*a*^3^ respectively.

**Figure 4:**
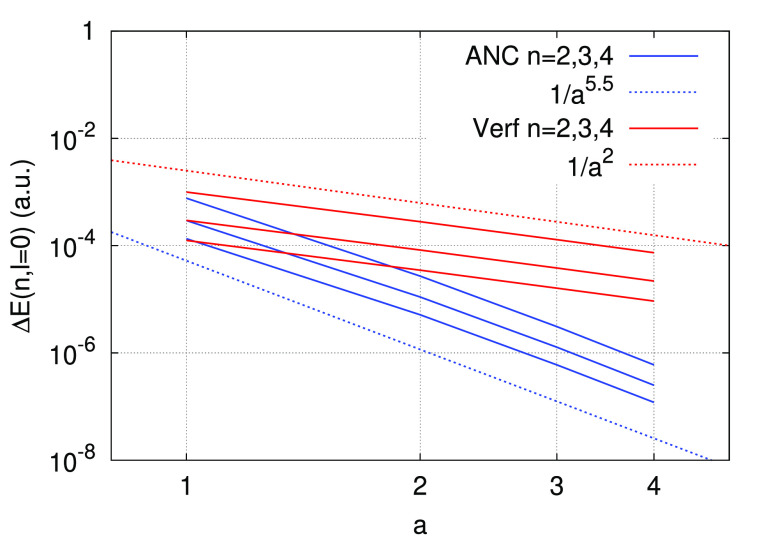
Decay of the error Δ*E*(*n*, *l* = 0) as a function of the parameter *a*, computed with an ANC potential (blue lines) and with a regularized potential −erf (*μr*)/*r* with μ = 10*a*. The top blue line corresponds to *n* = 2 and the bottom blue line to *n* = 4. The order of the red lines is similar. The blue and red dotted lines show the decay of a function ∝1/*a*^5.5^ and ∝1/*a*^2^, respectively.

In order to illustrate the importance of the norm conservation condition, we analyze the convergence of the eigenvalues of the *n* > 1, *l* = 0 solutions for the noninteracting hydrogen atom. While the *n* = 1 eigenvalue is exact by construction (*E*_1*s*_ = −1/2), higher eigenvalues for *n* = 2, 3, ... are affected by an error that decreases as the parameter *a* is increased to approach the exact Coulomb potential. We have computed the eigenvalues *E*(*n*, *l* = 0) for various values of *a* using a radial Schrödinger equation solver based on a finite difference representation of the Laplacian operator, resulting in a tridiagonal Hamiltonian matrix that is diagonalized using the LAPACK library. We find that the error in *E*(*n*, *l* = 0), defined as
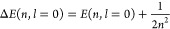
12decreases as the value of *a* is increased. The decay of the error follows approximately the power law 1/*a*^5.5^. We note that, as expected, the error in the eigenvalues becomes smaller for large *n*, since the amplitude of the corresponding hydrogenoid orbitals gets smaller near the nucleus, where the ANC potential deviates from the Coulomb potential. The rapid decrease of the error for increasing *a* is a consequence of the norm conservation condition, and plays a critical role in achieving convergence using reachable plane wave cutoffs. In order to illustrate the importance of the norm conservation condition, we show on Figure 3 the decay of Δ*E*(*n*, *l* = 0) for *n* = 2, 3, 4 computed with the ANC potential *V*(*a*, *b*, *r*) compared to the error obtained using *V*(*a*, *b* = 0, *r*) (i.e., a potential that reproduces the 1*s* eigenvalue exactly but does not satisfy the norm conservation condition). The decay of the error for *V*(*a*, *b* = 0, *r*) is slower than for the ANC potential, and only decreases approximately as 1/*a*^3^. We also compare the ANC potential with a simple regularized potential
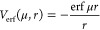
13that does *not* reproduce the 1*s* eigenvalue exactly and does *not* satisfy a norm conservation condition. The computed eigenvalues show an even slower decrease of the error, which decays approximately as 1/*a*^2^. Figure 4 shows the decay of the error in the *E*(*n*, *l* = 0) eigenvalues for *n* = 2, 3, 4 computed with the ANC potential and the potential −erf(*μr*)/*r* for μ = 10*a*.

Similar convergence results are found for the ANC potential for *l* = 1 and *l* = 2 (see Figure 1 in the Supporting Information). We note that the error in *E*(*n* = 2, *l* = 0) obtained with the ANC potential is smaller than 1 μHa for *a* = 4, and errors for *n* > 2 are even smaller.

#### 2.1.3 Scaling Relation

The ANC potential for arbitrary elements (*Z* > 1) can be derived simply from the *Z* = 1 potential using the following scaling relation:
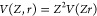
14where *V*(*r*) is the hydrogen atom ANC potential defined above. Under the transformation *r* → *Zr*, the Schrödinger equation for the hydrogen atom becomes
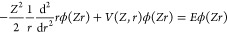
15It is easily verified that the function
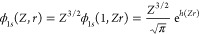
16is a solution of the scaled Schrödinger equation with eigenvalue
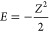
17The 1s function is a solution of the Schrödinger equation for the potential
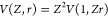
18We therefore define the ANC potential for *Z* > 1 as
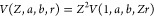
19

The noninteracting electronic structure for *Z* > 1 follows the expected behavior from the above scaling relation. The error in hydrogenoid eigenvalues scales as *Z*^2^. For *a* = 4, this changes the error in the *E*_2*s*_ eigenvalue from 0.64 μHa for H (Z = 1) to 63 μHa for Ne (Z = 10), and 205 μHa for Ar (Z = 18). These errors can be reduced by increasing the value of the *a* parameter.

## 3 Implementation

The use of a plane wave basis for all-electron calculations relies critically on an implementation of the plane wave method that is capable of scaling to very large plane wave energy cutoffs (e.g., of the order of 100 kRy). We use the Qbox code^24,25^ which was designed for large scale parallelism. Qbox can use large plane wave basis sets by distributing plane wave basis functions over thousands of processors. The use of a conventional reciprocal space preconditioner is effective, and the number of iterations needed to complete a calculation is similar to that of conventional pseudopotential calculations. Furthermore, Qbox includes the capability to change the plane wave cutoff on the fly during a calculation, which makes it possible to reach convergence gradually, in a way similar to the Full Approximation Scheme (FAS) used in multigrid methods.^26^ It also allows for changes of ionic potential during a calculation, which allows for systematic increase of the parameter *a* during a single calculation.

## 4 DFT Calculations

Having verified the transferability of ANC potentials to energies that differ from the 1s eigenvalue in the noninteracting case, we now proceed to demonstrate the accuracy of the AEPW approach in DFT calculations of atoms, solids, and liquids. In the application to DFT calculations, it is important to note that the regularized potential is defined independently of any choice of density functional, since it is taylored to reproduce the noninteracting problem. This allows for a comparison of density functionals in calculations using the same ANC potential and the same basis set. Examples of such comparisons are given below in calculations of the band structure of selected solids using the PBE^27^ and SCAN^28^ exchange-correlation functionals.

### 4.1 Atoms

We first test the AEPW approach on the simple problem of the DFT electronic structure of the hydrogen atom. Accurate results were obtained by Kotochigova et al.^29,30^ who used an atomic program based on a logarithmic radial mesh and achieved an accuracy better than 1 μHa for the Kohn–Sham energy. Their calculations used the Vosko-Wilk-Nusair (VWN) exchange-correlation functional^31^ and yielded a (spin-restricted) Kohn–Sham energy *E*_KS_ = −0.445671 Ha. In order to allow for a comparison with this reference result, we computed the Kohn–Sham energy of a single hydrogen atom placed in an FCC unit cell having a lattice parameter of 40 (a.u.). The use of this large unit cell makes the interaction between periodic replicas negligible on the scale of the errors considered. A systematic study of the convergence with respect to the parameter *a* and the plane wave energy cutoff is given in Table 1 where we show deviations Δ*E* from the AEPW value *E* = −0.44567052 Ha obtained using *a* = 20 (a.u.) and *E*_cut_ = 10 kRy, which we consider to be converged within 0.01 μHa. We note that this converged value agrees with the result of Kotochigova et al. within less than 1 μHa. This close agreement provides a remarkable verification of the two numerical methods involved in computing this number: on one hand, a solution of the Kohn–Sham equations on a radial logarithmic mesh, and on the other hand a plane wave calculation using a norm-conserving regularized potential. Table 1 shows that using *a* = 4 and *E*_cut_ = 1 kRy yields an error smaller than 10 μHa. Using *a* = 7 and a plane wave cutoff of 2 kRy yields an error of 1.04 μHa. The error can be further reduced by increasing *a*, with a corresponding increase of *E*_cut_. The minimum *E*_cut_ needed to converge the energy within 0.01 μHa grows proportionally to *a*^2^ and follows approximately the relation *E*_cut_ ≃ 0.025*a*^2^ where *E*_cut_ is expressed in kRy units. The values of *E*_cut_ given in Table 1 are sufficient to ensure a converged value of the energy within 0.01 μHa for the corresponding values of *a*. A more complete table showing additional values of *a* and *E*_cut_, and a contour plot of the logarithm of the error are available in the Supporting Information.

**Table 1:** Error in the DFT Ground State Energy of the Hydrogen Atom Computed Using the VWN Functional and Various Values of the Parameter *a* and Plane Wave Energy Cutoff *E*_cut_. The Values of *E*_cut_ Given in the Table Ensure a Converged Energy within 0.01 μHa for the Corresponding Value of *a*

*a*	*E*_cut_ (kRy)	Δ*E* (μHa)
4	1	9.00
5	1	3.97
6	1	1.97
7	2	1.04
8	2	0.59
9	3	0.36
10	4	0.22
11	4	0.15
12	4	0.10
13	5	0.07
14	5	0.05

In order to illustrate the behavior of the error for other atoms, we consider Be (Z = 4). Convergence of the KS VWN energy to within 1 μHa requires an increase of the value of *a*, and the value of *E*_cut_ must be increased correspondingly to reach convergence with respect to basis set size for a given *a*. We report in Table 2 the error in the energy with respect to the reference result −14.447209 (a.u.) given in ref (29), for various choices of *a* and *E*_cut_.

**Table 2:** Error in the DFT Ground State Energy of the Beryllium Atom Computed Using the VWN Functional and Various Values of the Parameter *a* and Plane Wave Energy Cutoff *E*_cut_

*a*	*E*_cut_ (kRy)	Δ*E* (μHa)
4	10	26
6	16	11
8	30	3.4
10	38	1.5

These results show that accurate energies can be obtained from AEPW calculations when increasing the parameter *a* and the plane wave cutoff, leading to errors of the order of 1 μHa. This accuracy criterion is however overly stringent, and useful information about physical quantities such as, e.g., band gaps, ionic forces, or stress tensor components can be obtained even if absolute energies are not converged to that degree. We show in the next section that for solids, such physical quantities can be computed with high accuracy, and most importantly that convergence can be tested systematically using the parameters *a* and *E*_cut_.

### 4.2 Solids

AEPW band structure calculations were carried out for diamond, silicon, MgO, and solid argon. We have computed the Kohn–Sham eigenvalues and band gaps at the high symmetry points of the Brillouin zone. The calculation of the stress tensor benefits from the simplicity of the plane wave basis, and requires no additional implementation compared to the standard method used in pseudopotential calculations. We use the approach of Focher et al.^18^ to ensure that a constant resolution is used when changing the unit cell size. All calculations are performed using the 10-point k-point set of Chadi and Cohen^32^ to sample the FCC Brillouin zone. We have verified in the case of pseudopotential calculations that using a 8 × 8 × 8 or 10 × 10 × 10 Monkhorst–Pack k-point set changes the band gaps by less than 0.005 eV.

### 4.3 Diamond

We have computed the band structure of diamond using the AEPW method with the PBE^27^ and SCAN^28^ exchange-correlation functionals. We use the experimental value of the FCC lattice constant *a*_FCC_ = 3.567 Å as reported by Haas et al.^33^ The main AEPW band gaps are reported in Table 3 for the PBE functional, and Table 4 for the SCAN functional. The value of the minimum band gap *E*_*g*_ is obtained using a quadratic fit to four eigenvalues along the Δ direction of the Brillouin zone in the range [0.30, 0.45](2π/*a*_FCC_). The minimum of the conduction band is found at *k* = 0.36(0, 0, 2π/*a*_FCC_) in all calculations reported.

**Table 3:** Energies (eV) of the Lowest Conduction Bands of Diamond at High-Symmetry Points of the BZ Referred to the Valence Band Maximum, and Minimum Energy Gap *E*_*g*_, Obtained Using the PBE Exchange-Correlation Functional and Various Values of the Parameter *a* and Plane Wave Energy Cutoff *E*_cut_

*a*	*E*_cut_ (kRy)	Γ	X	L	W	*E*_*g*_
2	6	5.600	4.770	8.467	10.626	4.138
3	6	5.600	4.775	8.468	10.627	4.142
4	6	5.600	4.778	8.468	10.627	4.145
2	10	5.600	4.770	8.468	10.626	4.138
3	10	5.600	4.775	8.468	10.627	4.142
4	10	5.600	4.775	8.468	10.627	4.143

**Table 4:** Energies (eV) of the Lowest Conduction States of Diamond at High-Symmetry Points of the BZ Referred to the Valence Band Maximum, and Minimum Energy Gap *E*_*g*_, Obtained Using the SCAN Exchange-Correlation Functional and Various Values of the Parameter *a* and Plane Wave Energy Cutoff *E*_cut_

*a*	*E*_cut_ (kRy)	Γ	X	L	W	*E*_*g*_
2	6	6.146	5.187	9.127	11.331	4.539
3	6	6.147	5.192	9.127	11.332	4.543
4	6	6.147	5.195	9.127	11.333	4.546
2	10	6.146	5.187	9.127	11.331	4.539
3	10	6.147	5.192	9.127	11.332	4.543
4	10	6.147	5.192	9.127	11.332	4.544

These results show that an accuracy of 0.01 eV is reached for *a* = 3 and *E*_cut_ = 6 kRy. Using larger values of *a* and *E*_cut_ lead to no appreciable change in the eigenvalues. FLAPW calculations by Doumont et al.^17^ find  = 4.14 eV and  = 4.54 eV in complete agreement with our AEPW results. A comparison of AEPW results with pseudopotential calculations using a SG15 optimized norm-conserving Vanderbilt (ONCV) pseudopotential^34^ and *E*_cut_ = 120 Ry gives a measure of the error introduced by the use of a pseudopotential. We find  = 4.17 eV and  = 4.48 eV, i.e., errors of 0.03 and 0.06 eV, respectively. In the case of the PBE calculation, the error is only due to the use of the pseudopotential approximation, while in the case of SCAN, an additional error comes from the fact that the SG15 pseudopotential was derived for the PBE functional.

The equilibrium lattice constant can be obtained by computing the stress tensor for various values of the lattice constant. The calculation of the stress tensor requires a larger plane wave cutoff than the one needed to converge band gaps. Using *E*_cut_ values up to 40 kRy, we verified that using *E*_cut_ = 15 kRy yields stress tensor components within 0.05 GPa of the fully converged value. We computed the PBE equilibrium lattice constant using *E*_cut_ = 15 kRy and *a* = 4. The stress tensor was computed for two values of the lattice constant, *a*_FCC_ = 3.5670 Å and *a*_FCC_ = 3.5825 Å. We use the confinement potential method of Focher et al.^18^ to ensure constant resolution of the plane wave basis as the cell volume is varied. The equilibrium lattice constant, corresponding to zero stress, was then obtained using the secant method, yielding the value 3.572 Å, in very good agreement with the FLAPW value reported by Haas et al. (3.575 Å). Using the same approach with the SCAN functional yields the value *a*_FCC_ = 3.552 Å. Tran et al.^35^ obtained the value 3.556 Å using an FLAPW non-self-consistent calculation based on PBE orbitals and density.

### 4.4 Silicon

We have computed the band structure of silicon using the AEPW approach with the PBE^27^ and SCAN^28^ density functionals, and compared results with existing reference data. We use plane wave energy cutoffs of 60 kRy and 80 kRy. The parameter *a* defining the ANC potential is varied between 3 and 4. AEPW eigenvalues change by less than 0.01 eV when changing the parameter *a* from 3 to 4, and when changing *E*_cut_ from 60 kRy to 80 kRy. In order to facilitate comparisons with other published work, we use the experimental lattice constant *a*_FCC_ = 5.430 Å reported by Haas et al.^33^ and used by other authors. The values of the conduction band eigenvalues relative to the valence band maximum are shown in Tables 5 and 6 The value of the minimum gap was computed by a quadratic fit to four values in the range [0.30, 0.45](2π/*a*_FCC_) along the Δ axis of the FCC Brillouin zone. The minimum is found at a value of *k* = (0.42, 0, 0)(2π/*a*_FCC_) in all calculations reported here.

**Table 5:** PBE Eigenvalues of the Lowest Conduction States of Silicon Referred to the Valence Band Maximum, Compared to SG15 Pseudopotential and with the LAPW Results of Reference (36)

	AEPW (this work)	SG15	FLAPW^36^
Γ	2.56	2.56	2.56
X	0.73	0.69	0.71
L	1.59	1.52	1.54
*E*_*g*_	0.59	0.56	0.47

**Table 6:** SCAN Eigenvalues of the Lowest Conduction States of Silicon Referred to the Valence Band Maximum, Compared to Pseudopotential Results Obtained with the SG15 Potentials Derived Using the PBE Functional

	AEPW (this work)	SG15
Γ	2.86	2.93
X	0.98	0.97
L	1.98	1.86
*E*_*g*_	0.83	0.83

Table 5 includes FLAPW results obtained by Betzinger et al.,^36^ which are in very good agreement with AEPW results at high-symmetry points of the BZ, the largest difference being 0.02 eV. The values of the minimum gap *E*_*g*_ on the other hand differ by 0.09 eV. The PBE minimum gap was also computed by Doumont et al.^17^ who used the WIEN2k FLAPW code and reported a value of 0.58 eV, in very good agreement with our AEPW value (0.59 eV). We also include in Table 5 the PBE eigenvalues computed using the SG15 pseudopotential^34^ with an 80 Ry plane wave cutoff. The comparison between the AEPW and SG15 values provides an estimate of the error caused by the use of the pseudopotential. The error for the gaps reported here is smaller than 0.07 eV.

We have repeated the above calculations using the SCAN functional. Results are shown in Table 6. In this case, the comparison between the AEPW and SG15 values provides an estimate of the error caused by the use of a pseudopotential derived using the PBE functional. This error is somewhat larger than the one due to the pseudopotential approximation alone, and affects the reported eigenvalues by less than 0.12 eV.

Doumont et al. also computed the value of the SCAN minimum gap, and obtained a value of 0.83 eV, in exact agreement with our AEPW value.

These results show that the AEPW approach reproduces reference results very accurately, both for a generalized gradient (GGA) and for a meta-GGA (SCAN) density functional.

### 4.5 Solid Argon

FCC argon provides an example of a system in which conduction states are delocalized, and thus require a basis set that properly describes multiple length scales. This can be achieved, e.g., in FLAPW calculations by adding localized orbitals to the APW basis with appropriately chosen energy parameters. In order to allow for a comparison with FLAPW results obtained by Michalicek et al.,^16^ we computed the band structure of FCC Ar using the LDA exchange-correlation functional. AEPW calculations of the LDA band structure of FCC Ar were performed using *a* = 3 and *E*_cut_ = 40kRy. Additional calculations with *a* = 4 and *E*_cut_ = 60 kRy confirmed that Kohn–Sham band gaps are converged within 0.01 eV. In order to allow for a comparison with the FLAPW results obtained by Michalicek et al.^16^ we use the same value of the experimental lattice constant reported in that paper (*a*_FCC_ = 9.93 (a.u.)). The AEPW results are shown in Table 7 and compared to the FLAPW+HDLO1x results of ref (16). The agreement between the two methods is excellent, with most deviations amounting to 0.01 eV and the largest being 0.04 eV. Michalicek et al. analyzed the convergence of the FLAPW method and noted that the addition of localized orbitals (LOs) to the FLAPW basis is essential to obtain accurate band energies. The addition of LO basis functions in ref (16) causes a downward shift of 1.87 eV in the lowest conduction band at the Γ point, bringing the result in close agreement with our results.

**Table 7:** LDA Eigenvalues of the Lowest Conduction States of FCC Ar Referred to the Valence Band Maximum, Compared to FLAPW+HDLOx1 Results of Reference (16)

	AEPW (this work)	FLAPW+HDLOx1^16^
Γ	8.25	8.21
X	10.86	10.85
L	11.08	11.07
W	11.92	11.92

We note that the FLAPW+HDLOx1 results treat core functions fully relativistically, valence functions using a scalar-relativistic approximation in the MT spheres, and use a nonrelativistic approach in the interstitial regions.^37^ This does not allow for a straightforward comparison of the results with the AEPW data which is nonrelativistic. However, on the basis of the results of ref (29) for atoms, we estimate that the effect of a scalar relativistic treatment on valence energy *differences* is small on the scale of errors considered here. These results show that the AEPW approach can reproduce accurate FLAPW results, even in cases where local orbitals must be included in the LAPW basis. The simplicity of the plane wave method guarantees a systematic convergence of the basis set for the description of both valence and conduction states.

### 4.6 MgO

The calculation of the AEPW band structure of MgO provides an example of validation of the pseudopotential approximation in a situation involving significant charge transfer. Pseudopotentials (in this case both for Mg and O) are typically derived to reproduce the electronic states of a neutral atom. In MgO, both Mg and O undergo a charge transfer of approximately two electrons. It is important to quantify the effect of the pseudopotential approximation, which assumes no polarizability of the core shells and additivity of the exchange-correlation potential. We have computed the AEPW band structure of MgO in the rocksalt structure using the PBE exchange-correlation functional and a sequence of ANC potentials, with *a* up to 3 (a.u.) for Mg and up to 4 (a.u.) for O, with a plane wave cutoff *E*_cut_ = 30 kRy. We have verified that eigenvalues change by less than 0.01 eV when increasing the plane wave cutoff to 40 kRy. In order to allow for comparison with other published results, we use the experimental lattice constant *a*_FCC_ = 4.207 Å reported by Haas et al.^33^Table 8 compares AEPW results with the FLAPW results reported by Betzinger et al.^36^ and Schlipf et al.,^38^ as well as PAW results obtained by Paier et al.^39^

**Table 8:** PBE Eigenvalues (eV) of the Lowest Conduction States of FCC MgO Referred to the Valence Band Maximum, Compared to LAPW, PAW, and Pseudopotential (SG15) Results

	AEPW (this work)	FLAPW^36^	FLAPW^38^	PAW^39^	SG15
Γ	4.83	4.84	4.77	4.75	4.80
X	9.14	9.15	9.14	9.15	9.19
L	7.94	8.01	7.93	7.91	7.95

The AEPW results show a very good agreement with the FLAPW and PAW results, with a maximum deviation of 0.08 eV. The SG15 results agree with the AEPW results within 0.05 eV. This provides an estimate of the error due to the use of the pseudopotential approximation. In spite of a large charge transfer, the SG15 pseudopotential appears to be transferable to configurations that differ from the atomic situation, both in terms of symmetry and charge state. In a more sensitive test, we have also verified that the transferability of the SG15 potentials extends to the calculation of the stress tensor. The σ_*xx*_ component of the residual stress tensor at the experimental lattice constant was computed with the SG15 potential using *E*_cut_ = 100 Ry, yielding  = 5.58 GPa, while the corresponding AEPW value is 5.51 GPa.

### 4.7 Liquid Water

**Figure 5:**
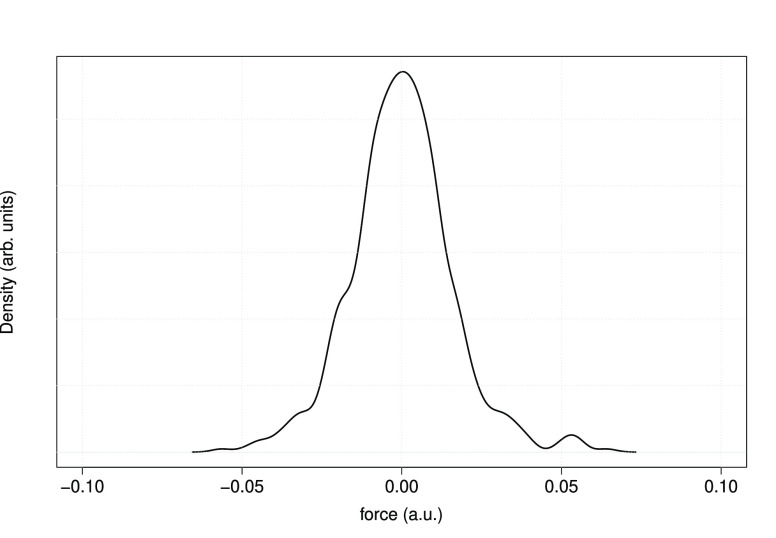
Distribution of ionic forces in a 64-molecule H_2_O snapshot, computed using the AEPW approach with *E*_cut_ = 30 kRy. The Gaussian kernel bandwidth used is 0.003 (a.u.).

In this example, we compute ionic forces in a 64-molecule sample of water using the PBE density functional. We use a representative snapshot extracted from a molecular dynamics trajectory taken from the PBE400 data set.^40,41^ AEPW calculations were carried out with *E*_cut_ = 30 kRy and *a* = 8 for hydrogen, and *a* = 3 for oxygen. We verified that changing *E*_cut_ to 25 kRy affects ionic forces by less than 10^–5^ (a.u.), and consider the results obtained at 30 kRy to be accurate within that tolerance. Figure 5 shows a Gaussian kernel density estimate of the distribution of AEPW ionic forces. Forces on all 192 atoms in the *x*, *y*, and *z* directions are included in the data set, for a total of 576 values. All components of the forces fall within the range [−0.06, 0.06] (a.u.).

**Figure 6:**
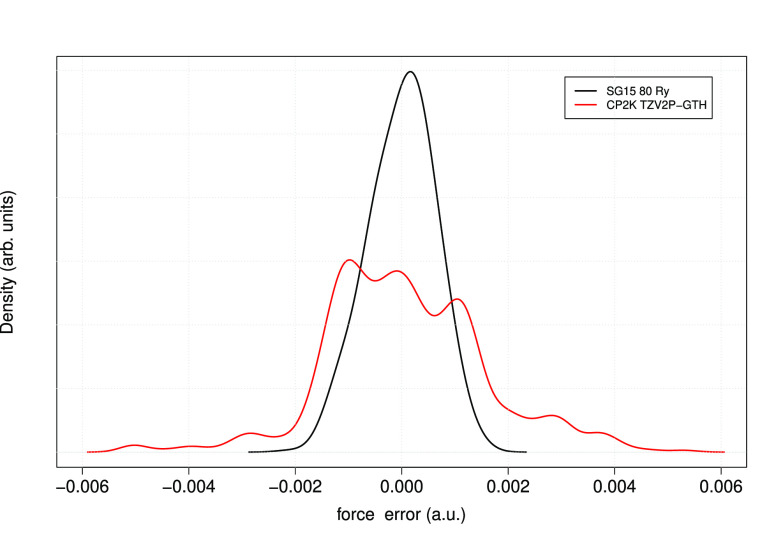
Distribution of the error in ionic forces computed using an SG15 pseudopotential and a TZV2P-GTH basis set, referred to the AEPW results. The Gaussian kernel bandwidth used is 0.00025 (a.u.).

In a first comparison, we validate the use of the pseudopotential approximation by comparing AEPW forces with those obtained with SG15 pseudopotentials, using *E*_cut_ = 80 Ry. Similarly, for a comparison with a calculation based on atom-centered basis functions, we have used the CP2K program^42^ (version 7.1) and the TZV2P-GTH combination of Gaussian basis set and pseudopotentials to evaluate ionic forces on the same atomic configuration. We used a plane wave cutoff of 400 Ry for the evaluation of the charge density in CP2K. We show in Figure 6 the distribution of the deviation of ionic forces with respect to the AEPW forces, for both the SG15 pseudopotential calculation and for the TZV2P-GTH calculation. Both calculations show a small error in ionic forces—most errors being smaller than 0.002 (a.u.)—with the TZV2P-GTH forces showing somewhat larger deviations from the reference AEPW results.

The above calculations provide an example of use of AEPW calculations to validate approximations used in other electronic structure methods. Similar validations can be performed using, e.g., other density functionals such as SCAN to estimate errors due to the use of PBE pseudopotentials, or to assess the effect of the choice of another atom-centered basis set.

## 5 Discussion

The above results show that the AEPW approach can reach an accuracy comparable to the most accurate FLAPW methods in a number of periodic solids. Convergence of results can be systematically tested by increasing the parameter *a* while simultaneously increasing *E*_cut_ to achieve full convergence for each value of *a*. The absence of any other parameters in the calculation—apart from k-point sampling—leads to a high confidence in the accuracy of the results. In particular, the completeness of the basis set can be reached systematically for both occupied and empty orbitals without any prior knowledge of the electronic structure of the atoms. Another key feature of the plane wave method is the possibility of enforcing constant resolution of the basis set while varying unit cell parameters.^18^ It is generally observed that convergence of the stress tensor requires a higher plane wave cutoff and larger values of *a* than convergence of band gaps, which benefit from cancellation of errors in the absolute eigenvalues. Nevertheless, the stress tensor computed with an appropriately increased *E*_cut_ yields accurate values of equilibrium lattice constants. Importantly, the AEPW approach allows for the direct calculation of the stress tensor and does not rely on a fit of the energy to an equation of state for the calculation of the equilibrium lattice constant. This is particularly relevant in systems of lower symmetry in which the unit cell is described by multiple parameters, which make the fitting procedure impractical. Our last example (liquid water) also shows that an AEPW calculation can be used to test the accuracy of ionic forces. The validity of the pseudopotential approximation, and/or the use of a localized basis set, can be tested quantitatively.

The AEPW approach is expected to enable precise comparisons between different exchange-correlation functionals, without the additional uncertainty associated with the construction of a pseudopotential appropriate for a given density functional. It also avoids the need to rely on approximations in the FLAPW method such as described in ref (17) where the self-consistent computation of localized orbitals added to the basis may not be feasible with complex, e.g., meta-GGA, functionals.

AEPW calculations are straightforward but computationally expensive, particularly in terms of memory usage, due to the large plane wave basis used. The calculations presented here were made possible by the scalability of the Qbox code that distributes simultaneously plane wave basis functions, bands, and k-points to different processor partitions. While the smaller calculations (e.g., diamond band structure) fit on a moderate-size cluster, the larger ones (e.g., (H_2_O)_64_ and FCC Ar) used up to 512 nodes of the Theta Intel-Cray XC40 computer installed at Argonne National Laboratory.

## 6 Conclusions

We have demonstrated the feasibility of all-electron, plane wave electronic structure calculations. Calculations of the electronic structure of atoms and periodic solids show that accurate values of energies, ionic forces, stress tensor, and band gaps can be obtained. The method relies on the use of an analytic, norm-conserving, regularized potential that replaces the Coulomb potential describing the electron–ion interaction. The calculations presented make use of a scalable implementation of the plane wave method that can accommodate large plane wave energy cutoffs, up to 80 kRy in the examples considered. Fast convergence of the self-consistent iterations is achieved by gradually increasing the plane wave energy cutoff during the calculation, in a process similar to the Full Approximation Scheme used in multigrid methods. The simplicity of the plane wave method makes it an appealing approach when implementing complex density functionals, such as meta-GGA functionals. Quantities such as ionic forces or stress tensors are readily available in a conventional implementation without additional work. AEPW calculations also allow for the validation of the approximation in which a pseudopotential derived using a given exchange-correlation functional is then used with another functional. An example of such validation using the SCAN functional with PBE-derived potentials was presented for diamond and silicon. The extension of the AEPW method to include relativistic effects is under development.

## Supporting Information Available

Tables of the value of the *b* parameter, additional convergence data and figures (PDF)
