# Supplementary material for: All-Electron Plane-Wave Electronic Structure Calculations
Source: J Chem Theory Comput. 2023 Feb 9;19(4):1300–9. doi: 10.1021/acs.jctc.2c01191 (PMC9979607; doi:10.1021/acs.jctc.2c01191)
Supplement: Supplementary file 1 — ct2c01191_si_001.pdf [file ct2c01191_si_001.pdf]

# Supporting information for:

# All-electron plane-wave electronic structure calculations

François Gygi*

*Department of Computer Science, University of California Davis, Davis, CA95616 USA*

E-mail: fgygi@ucdavis.edu.

Table S1: Values of the parameter $b$ that satisfy the norm conservation condition for a given value of the parameter $a$. These values can be used for any value of $Z$.

| $a$ (a.u.)$^{-1}$ | $b$ (a.u.) |
|---|---|
| 1 | -3.6442293856e-01 |
| 2 | -1.9653418982e-01 |
| 3 | -1.3433604753e-01 |
| 4 | -1.0200558466e-01 |
| 5 | -8.2208091118e-02 |
| 6 | -6.8842555167e-02 |
| 7 | -5.9213652850e-02 |
| 8 | -5.1947028250e-02 |
| 9 | -4.6268559218e-02 |
| 10 | -4.1708913494e-02 |
| 11 | -3.7967227308e-02 |
| 12 | -3.4841573775e-02 |

*To whom correspondence should be addressed

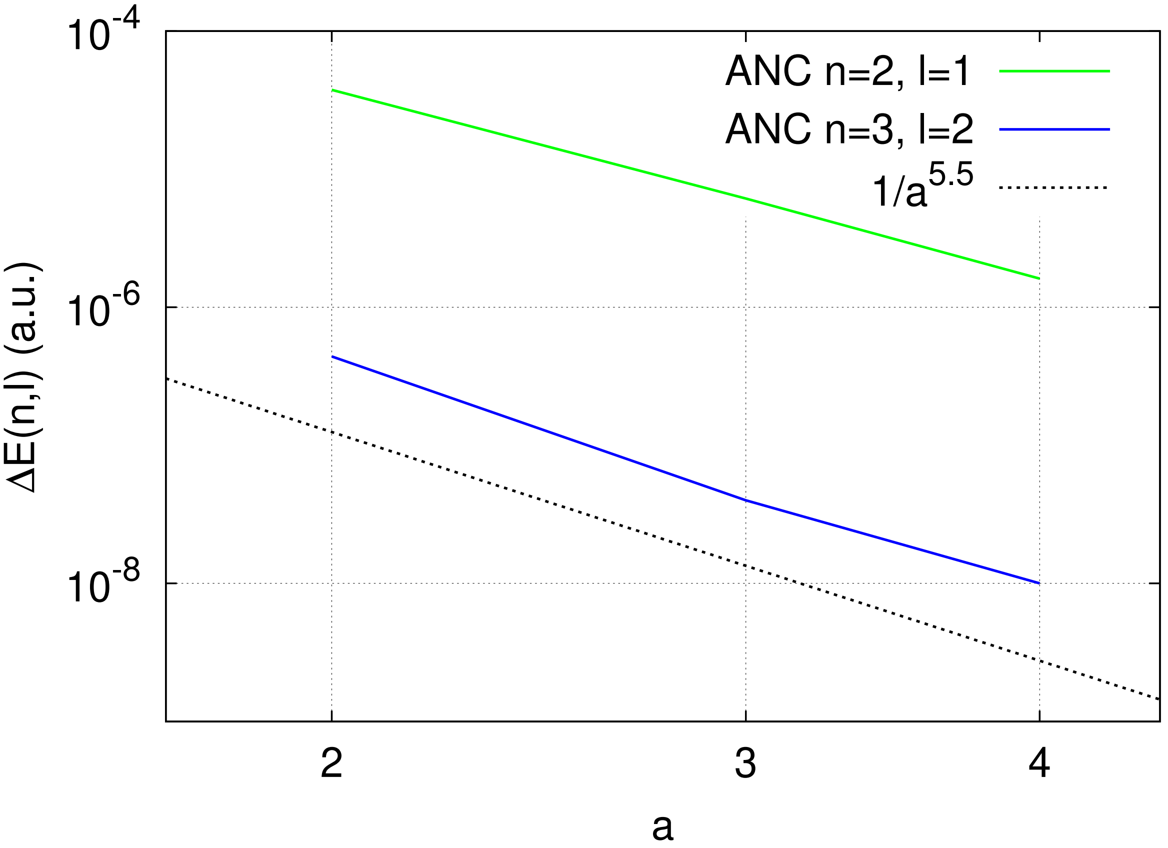


Figure S1: Decay of the non-interacting eigenvalue error $\Delta E(n = 2, l = 1)$ (green line) and $\Delta E(n = 3, l = 2)$ (blue line) as a function of the parameter $a$, computed with an ANC potential. The dotted line shows the decay of a function $\propto 1/a^{5.5}$.

Table S2: Ground state energy $E(a, E_{\text{cut}})$ of the hydrogen atom computed using the VWN functional, $a \in [4, 14]$ (a.u.)$^{-1}$, $a_{\text{FCC}} = 40$ (a.u.) for various values of $E_{\text{cut}}$.

| $a$ (a.u.)$^{-1}$ | E(2 kRy) | E(3 kRy) | E(4 kRy) | E(5 kRy) | E(6 kRy) |
|---|---|---|---|---|---|
| 4 | -0.44566153 | -0.44566153 | -0.44566153 | -0.44566153 | -0.44566153 |
| 5 | -0.44566656 | -0.44566656 | -0.44566656 | -0.44566656 | -0.44566656 |
| 6 | -0.44566859 | -0.44566859 | -0.44566859 | -0.44566859 | -0.44566859 |
| 7 | -0.44566949 | -0.44566949 | -0.44566949 | -0.44566949 | -0.44566949 |
| 8 | -0.44566994 | -0.44566994 | -0.44566994 | -0.44566994 | -0.44566994 |
| 9 | -0.44567016 | -0.44567017 | -0.44567017 | -0.44567017 | -0.44567017 |
| 10 | -0.44567023 | -0.44567030 | -0.44567031 | -0.44567031 | -0.44567031 |
| 11 | -0.44567016 | -0.44567038 | -0.44567038 | -0.44567038 | -0.44567038 |
| 12 | -0.44566991 | -0.44567040 | -0.44567043 | -0.44567043 | -0.44567043 |
| 13 | -0.44566946 | -0.44567038 | -0.44567045 | -0.44567046 | -0.44567046 |
| 14 | -0.44566885 | -0.44567031 | -0.44567046 | -0.44567048 | -0.44567048 |

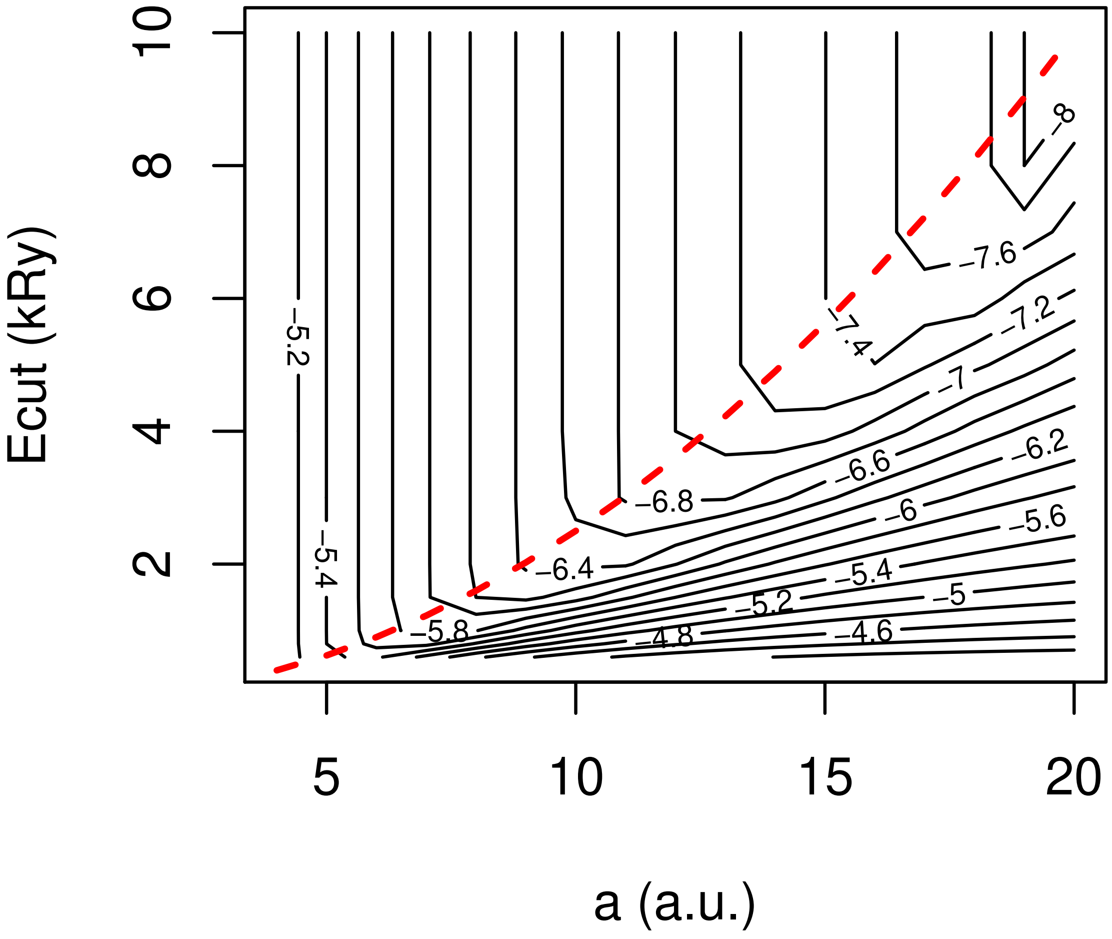


Figure S2: Contour plot of the base-10 logarithm of the error in the Kohn-Sham ground state energy of the hydrogen atom (a.u.) as a function of the parameter $a$ and the energy cutoff $E_{\mathrm{cut}}$. The dashed red line shows the relation $E_{\mathrm{cut}}$ (kRy) = 0.025 $a^2$.

Table S3: Ground state energy of the hydrogen atom computed using the VWN functional, $a = 12$ (a.u.)$^{-1}$, $a_{\text{FCC}} = 54$ (a.u.) for various values of $E_{\text{cut}}$.

| $E_{\text{cut}}$ (kRy) | E (a.u.) |
|---|---|
| 2 | -0.44567010 |
| 3 | -0.44567022 |
| 4 | -0.44567027 |
| 5 | -0.44567027 |
| 6 | -0.44567027 |

Table S4: PBE band gaps of diamond computed with $E_{\text{cut}} = 6$ kRy and $a = 4$ (a.u.)$^{-1}$, using the Chadi-Cohen 10 k-point set (CC) and the Monkhorst-Pack $8 \times 8 \times 8$ set (MP) for Brillouin zone sampling.

| | 10pt (CC) | 8x8x8 (MP) |
|---|---|---|
| Γ | 5.6003 | 5.5981 |
| X | 4.7776 | 4.7757 |
| L | 8.4679 | 8.4645 |
| W | 10.6274 | 10.6237 |
| $E_g$ | 4.1446 | 4.1426 |

Table S5: PBE stress tensor component $\sigma_{xx}$ of diamond computed with $E_{\text{cut}} = 6$ kRy and $a = 4$ (a.u.)$^{-1}$, using the Chadi-Cohen 10 k-point set (CC) and the Monkhorst-Pack $8 \times 8 \times 8$ set (MP) for Brillouin zone sampling.

| | 10pt (CC) | 8x8x8 (MP) |
|---|---|---|
| $\sigma_{xx}$ (GPa) | -1.8423 | -1.7817 |
